# International Forum on Visceral Myopathy 2024: Advances in the Knowledge of the Disease

**DOI:** 10.1111/nmo.70317

**Published:** 2026-04-14

**Authors:** Pascal De Santa Barbara, Isabella Ceccherini, Robert O. Heuckeroth, Carlo Di Lorenzo, Maria M. Alves, Arthur Beyder, Osvaldo Borrelli, Ludovica Cacopardo, Rachel Ceron, Jihong Chen, Federica Chiappori, Antonio Contessa, Roberto De Giorgio, Antonella Diamanti, Maria Grazia Faticato, Sandrine Faure, Sohaib K. Hashmi, Jan D. Huizinga, Raj P. Kapur, Cecile Lambe, Laurence Lancon, Ryan McManigal, Hayat Mousa, Alessandro Palmitelli, Victor Perreaux, Alessio Pini Prato, Michela Pitto, Elisa Proietti, John Rendu, Anna Rybak, Kenton M. Sanders, Sabah Sardar, Vincenzo Stanghellini, Renato Tambucci, Nikhil Thapar, Pieter Vanden Berghe, Massimo Vassalli, Qianqian Wang, Michael Wangler, Sharon Wolfson, Almira Zada, Jiliang Zhou, Federica Viti

**Affiliations:** ^1^ Physiology and Experimental Medicine of the Heart and Muscles (PhyMedExp), University of Montpellier INSERM, CNRS Montpellier France; ^2^ UOSD Aggregation Area of the Research Laboratories, IRCCS Giannina Gaslini Institute Genoa Italy; ^3^ Department of Pediatrics, Perelman School of Medicine University of Pennsylvania Philadelphia Pennsylvania USA; ^4^ The Children's Hospital of Philadelphia Research institute Philadelphia Pennsylvania USA; ^5^ Nationwide Children's Hospital Columbus Ohio USA; ^6^ Department of Clinical Genetics Erasmus University Medical Center‐Sophia Children's Hospital Rotterdam the Netherlands; ^7^ Department of Pediatric Surgery Erasmus University Medical Center‐Sophia Children's Hospital Rotterdam the Netherlands; ^8^ Enteric Neuroscience Program (ENSP), Division of Gastroenterology and Hepatology, Department of Medicine Mayo Clinic Rochester Minnesota USA; ^9^ Department of Physiology and Biomedical Engineering (BMEP) Mayo Clinic Rochester Minnesota USA; ^10^ Neurogastroenterology & Motility Unit, Department of Gastroenterology Great Ormond Street Hospital London UK; ^11^ Department of Information Engineering and Research Center ‘E. Piaggio’ University of Pisa Pisa Italy; ^12^ Farncombe Family Digestive Health Research Institute, Department of Medicine McMaster University Hamilton Ontario Canada; ^13^ National Research Council, Institute for Biomedical Technologies (CNR‐ITB) Milano Italy; ^14^ Uniti per la PIPO‐Patient Advocacy Organization Brescia Italy; ^15^ Department of Translational Medicine University of Ferrara Ferrara Italy; ^16^ Digestive Diseases and Nutritional Rehabylitation Pediatric Hospital Bambino Gesù Rome Italy; ^17^ Pediatric Surgery, IRCCS Giannina Gaslini Institute Genoa Italy; ^18^ Department of Laboratory Medicine and Pathology Seattle Children's Hospital and University of Washington Seattle Washington USA; ^19^ Department of Pediatric Gastroenterology and Nutrition, Hospital Necker‐Enfants Malades Université Paris Cité Paris France; ^20^ Association des POIC—Patient Advocacy Organization Marseille France; ^21^ World Visceral Myopathy Foundation Dallas Texas USA; ^22^ Poic e dintorni APS—Patient Advocacy Organization Rome Italy; ^23^ Nantes Université, Inserm, TENS UMR1235, The Enteric Nervous System in Gut and Brain Diseases, IMAD Nantes France; ^24^ Umberto Bosio Center for Digestive Diseases The Children Hospital, AOU SS Antonio e Biagio e Cesare Arrigo Alessandria Italy; ^25^ Institute of Biophysics, National Research Council Genoa Italy; ^26^ U.O.C. Di.P.S IRCCS Istituto Giannina Gaslini Genoa Italy; ^27^ Department of Internal Medicine and Medical Specialties University of Genoa Genova Italy; ^28^ Univ. Grenoble Alpes, Inserm, U1216, CHU Grenoble Alpes, Grenoble Institute Neurosciences Grenoble France; ^29^ Department of Physiology and Cell Biology, Reno School of Medicine University of Nevada Reno Nevada USA; ^30^ James Watt School of Engineering University of Glasgow Glasgow UK; ^31^ Alma Mater Studiorum—Università di Bologna Bologna Italy; ^32^ Gastroenterology and Nutrition Unit Bambino Gesù Children's Hospital IRCCS Rome Italy; ^33^ Stem Cell and Regenerative Medicine, GOS Institute of Child Health, University College London London UK; ^34^ Gastroenterology, Hepatology and Liver Transplant Queensland Children's Hospital Brisbane Queensland Australia; ^35^ School of Medicine, University of Queensland, Centre for Child Nutrition Research Queensland University of Technology Brisbane Queensland Australia; ^36^ Laboratory for Enteric NeuroScience (LENS), TARGID, KU Leuven University of Leuven Leuven Belgium; ^37^ Institute for Stem Cell Biology and Regenerative Medicine Stanford University School of Medicine Stanford California USA; ^38^ Department of Molecular and Human Genetics, Baylor College of Medicine Houston Texas USA; ^39^ Jan and Dan Duncan Texas Children's Neurological Research Institute Houston Texas USA; ^40^ Department of Biomedical Sciences Faculty of Medicine Universitas Padjadjaran Bandung Indonesia; ^41^ Department of Pharmacology, Toxicology & Neuroscience LSU Health Shreveport Shreveport Louisiana USA

**Keywords:** ACTG2, CIPO and PIPO, parenteral nutrition, smooth muscle cells, visceral myopathy

## Abstract

**Background:**

Visceral myopathy (VSCM) is an ultra‐rare life‐threatening condition characterized by severe impairment of gastrointestinal (GI), genitourinary, and uterine smooth muscle. This disorder represents a significant clinical challenge due to variable presentation and the lack of standardized diagnostic and therapeutic protocols.

**Methods:**

To discuss advances in the field, scientists and clinicians with a special interest in VSCM met in Arenzano, Genova, Italy in October 2024 for the second International Forum on Visceral Myopathy 2024 (IFVM2024) (https://ifvm2024.ge.ibf.cnr.it/).

**Key Results:**

As in the previous edition of the event (https://poic‐e‐dintorni.org/efvm‐2022/), attendees included clinicians and researchers from around the world who study this disease, representatives from support organizations, patients affected by VSCM and their families, and companies that co‐funded the event. The present manuscript aims to summarize knowledge shared during the IFVM2024 conference, thus providing an updated state‐of‐the‐art summary of VSCM biology and disease management.

**Conclusions:**

Here, we pay particular attention to the epidemiology of the disease, histopathology, genetics, novel treatments, advances in molecular and cell biology, experimental models, and the lived experiences and impact of this disorder on families.

## Abstract (highlights)

**Plain Language Summary:**

Visceral myopathy, the myogenic form of chronic intestinal pseudo‐obstruction, is an extremely rare, life‐threatening condition characterized by severe impairment of gastrointestinal, genitourinary and uterine smooth muscle. Genetic testing uncovers a monogenic cause in about 60% of affected individuals. Current therapy is largely supportive (through enteral or parenteral nutrition and surgery in selected cases), also due to the still scarce knowledge on the molecular mechanisms underlying the pathology. A collaborative international effort among researchers, clinicians and stakeholders (especially parent advocacy organizations) is ongoing, and it is crucial to create consensus on possible new diagnostic and therapeutic strategies, as well as to provide patient cohorts of adequate size for retrospective and prospective studies.

**Key Points:**

- Visceral myopathy, the myogenic form of chronic intestinal pseudo‐obstruction, is an extremely rare, life‐threatening condition characterized by severe impairment of gastrointestinal, genitourinary, and uterine smooth muscle. Diagnosis may be made by symptoms and imaging tests supported by manometry suggesting an underlying visceral myopathy. Genetic testing uncovers a monogenic cause in about 60% of affected individuals. Histopathology of muscle layers may be normal or non‐specific, although significant changes may also be seen. Current therapy is largely supportive (through enteral or parenteral nutrition and surgery in selected cases), while more definitive/curative therapies directed at specific etiopathogenetic mechanisms are lacking.
- Research on visceral myopathy is unveiling the molecular mechanisms that cause this disease, developing novel and useful models, and providing insight into possible pharmaceutical approaches to treat this devastating condition.
- A collaborative international effort among researchers, clinicians, and stakeholders, especially parent advocacy organizations (PAOs), is needed to create consensus on possible new diagnostic and therapeutic strategies, as well as to provide patient cohorts of adequate size for retrospective and prospective studies.

## Graphical abstract

As an orphan disease, where treatment is not curative and diagnosis is often slow, VSCM represents a serious health and social problem requiring research efforts in several directions.

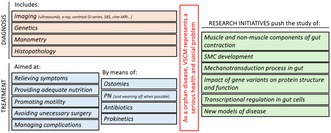

## 1 Introduction

Visceral myopathy (VSCM) is defined by weakness of bowel, bladder and uterine smooth muscle. Bowel smooth muscle weakness leads to ineffective propulsive contractions, inadequate mixing of luminal contents with digestive enzymes, delayed intraluminal aboral movement, very slow transit, and dilated bowel. Symptoms include massive abdominal distension, vomiting, constipation, and inability to survive or thrive solely on oral nutrition. This bowel problem in adults is called myopathic chronic intestinal pseudo‐obstruction (mCIPO). Pediatric chronic intestinal pseudo‐obstruction (PIPO) is currently used to designate childhood onset CIPO. CIPO may also be caused by problems with the enteric nervous system, extrinsic autonomic nervous system, interstitial cells of Cajal, or other cells that affect bowel motility. In VSCM, bladder muscle weakness causes bladder enlargement (megacystis) and poor bladder emptying. Uterine muscle weakness may cause problems during delivery. Smooth muscle dysfunction in the fetal bowel may be impaired causing a “microcolon”, as part of a syndrome called megacystis microcolon intestinal hypoperistalsis syndrome (MMIHS). Here we review discussions at the 2nd International Forum on Visceral Myopathy 2024 (IFVM2024) (https://ifvm2024.ge.ibf.cnr.it/), which highlighted state‐of‐the‐art knowledge and issues that require further research.

## 2 Epidemiology and Diagnosis of VSCM

### 2.1 Epidemiology

VSCM and related clinical phenotypes are considered extremely rare, although epidemiological data remain scant. A national survey of members of the North American Society of Pediatric Gastroenterology and Nutrition, published in 1988, suggested 87 children with CIPO were born in the United States every year (~22.2 affected per million live births) [1, 2]. Only 12 of these 87 children had full‐thickness bowel biopsies and 4 biopsies showed smooth muscle degeneration. A Japanese nationwide survey published in 2014 revealed a CIPO prevalence of 3.7 per 1 million, but no data were provided on the proportion of mCIPO [3]. Data from the United Kingdom, where a national service for the diagnosis of CIPO was established at Great Ormond Street Hospital (GOSH) in 2012, identified 114 children with CIPO in 11 years (~13 affected per million live births), with VSCM accounting for ~15% of all CIPO cases (~2 per million live births) [4, 5]. In other series, referral bias could impact statistics [6, 7]. In France, approximately 50 children receive home parenteral nutrition due to CIPO, suggesting a prevalence of about 1 in 1.3 million with about half due to *ACTG2* variants (unpublished data). Overall, available data suggest an estimated incidence of CIPO of ~1 per 60,000 live births and of VSCM of ~1 per 400,000 live births. New international data would be valuable, especially since genetic testing may now facilitate mCIPO diagnosis. Furthermore, data on adult prevalence of mCIPO are needed since adults may show milder disease phenotypes, which could provide valuable insight into the pathophysiology of VSCM. Genetic sequencing of adults with suspected CIPO would also be valuable, but genetic testing is not currently standard‐of‐care for adults.

Considering all types of CIPO, most studies indicate an equal sex incidence or slight female preponderance. For example, of 62 CIPO cases identified in the Japanese nation‐wide survey, 55% were females, in keeping with adult studies [8, 9, 10, 11]. A systematic literature review for pediatric MMIHS cases, however, revealed that 70.5% of patients were female, in keeping with 2 recent studies from Japan (nation‐wide surveys) where the proportion of female people with MMIHS was 84% and 68% respecively [12, 13]. This suggests that VSCM may be more common among females, especially in children.

### 2.2 Clinical Manifestation and Diagnosis

VSCM can manifest at different ages including prenatal, newborn, childhood, and adult presenting symptoms. Mechanisms that explain these differences in disease onset and severity need to be elucidated, but if understood could suggest new therapeutic targets. The most common manifestations of mCIPO include recurrent sub‐obstructive episodes with acute (or chronic) abdominal distension, vomiting; in addition, bowel habit changes (constipation or diarrhea), poor weight gain and malnutrition, as consequences of bowel smooth muscle weakness and impaired intestinal transit, can be identified. In some VSCM cases, microcolon is present [14]. Small intestinal bacterial overgrowth (SIBO) symptoms may occur because of slow transit of luminal contents through the dilated small bowel and can cause diarrhea, flatulence, bloating, and abdominal pain. Bladder enlargement with urinary retention, hydronephrosis, and vesico‐ureteral reflux are common manifestations of urinary tract smooth muscle weakness [15, 16]. In the United Kingdom VSCM cohort, almost all cases for which information is available had urinary tract involvement, especially megacystis (bladder enlargement; Table 1), and also in the Italian cohort [17, 18]. Nevertheless, clear information on the prevalence of such symptoms is not always available. Impaired uterine smooth muscle contraction may cause problems with the progression of labor and increase the risk of postpartum hemorrhage. Prenatal CIPO most often presents with megacystis and hydronephrosis (88%), polyhydramnios (34%), and gastric distension (10%) but rarely with dilated bowel [8]. In newborns, CIPO may present with volvulus as a manifestation of intestinal malrotation which occurs in ~25% of people with mCIPO (compared to 1 out of 200–500 live births) [19, 20]. The frequency of abdominal pain associated with VSCM is controversial: in the UK experience, only 8% of children with VSCM caused by *ACTG2* variants commonly reported abdominal pain (see Section 3.1).

**TABLE 1:** ACTG2 variants and summary of gastrointestinal tract involvement, treatment and nutrition.

Variant	*N*	Onset age (months)	GI involvement	Treatment	Histopathology (full thickness biopsies)	Age at stoma	Nutrition	Other notes
c.769C>T (p.R257C)	4	Birth 1 6 12	Stomach/SB (2), pan‐enteric (2)	Ileostomy (4), gastrostomy (4), PEG‐J (2); Ladd's procedure (1), colectomy (1)	Normal (1), Myopathic changes (2), n/a (1)	1.9–2.9 years	100% oral (1) 100% PN (1) 100% jejunal (1) 30% oral, 70% PN (1)	Megacystis (4) craniosynostosis, prematurity (1)
c.770G>A (p.R257H)	2	Birth (2)	Stomach/SB (1), pan‐enteric (1)	Ileostomy (2), Ladd's procedure (1)	Normal (2)	2.5–4.5 years	Partial PN/enteral (1) 100% PN (1)	Megacystis (1)
c.533G>T (p.R178L)	1	Birth	n/a	Ladd's, SB resection, ileostomy	Normal	1 month	100% PN	Megacystis, microcolon
c.532C>T (p.R178C)	1	Birth	n/a	Ladd's, ileostomy, gastrostomy	n/a	1 month	100% PN	Malrotation, megacystis, microcolon
c.533G>A(p.R178H)	1	1	Stomach, SB	Ileostomy, hemicolectomy	n/a	1 month	100% PN	
c.632G>A (p.R211G)	1	1	Pan‐enteric	Duodeno‐jejunostomy, gastrostomy, ileostomy	Normal	15 years	100% PN	Affected paternal grandmother
c.119G>A (p.R40H)	1	6	Pan‐enteric	Ileostomy, gastrostomy	n/a	4.5 years	100% PN	Affected mother, brother. Bladder dysfunction
c.593G>T(p.G198V)	1	Birth	n/a	Ileostomy, gastrostomy	n/a	1.5 years	100% PN	Megacystis
c.338C>G(p.P113R)	1	84	Pan‐enteric	Ileostomy	Normal	15 years	100% enteral	

Abbreviations: n/a, not available; PEG‐J, percutaneous endoscopic gastro‐jejunostomy; PN, parenteral nutrition; SB, small bowel.

Remarkably, symptom severity varies dramatically over time in single affected individuals. Specifically, people with VSCM typically have episodes where symptoms become much more severe separated by periods of relative improvement. These episodes of increased symptom severity may be associated with infection, poor nutritional status, anesthesia, surgery, partial mechanical obstruction (ostomy dysfunction, adhesions, volvulus), dietary factors, changes in gut microbes, and changes in medicines (and perhaps other factors). The variable severity of CIPO symptoms contributes to the challenge of making an accurate and timely diagnosis or predicting long‐term outcomes.

#### 2.2.1 Imaging for Anatomical and Functional Gut Assessment

A diagnostic algorithm recommended by the European Society of Pediatric Gastroenterology, Hepatology and Nutrition (ESPGHAN) emphasizes the importance of ruling out mechanical obstruction and confirming GI dysmotility, while ensuring that treatable causes are excluded [15]. Radiological tests are commonly used in VSCM and PIPO due to their minimal invasiveness and wide accessibility. These imaging tests may exclude mechanical obstruction, support a diagnosis of VSCM, monitor disease progression, or direct surgical management. Moreover, radiology and imaging can provide information on gut function.

Key imaging approaches and limitations follow:

– Antenatal ultrasound can detect prenatal signs (most commonly a dilated urinary tract/bladder) in 20%–50% of PIPO cases and is especially useful in VSCM where most cases, if not all (e.g., MMIHS cases), show prenatal signs of VSCM [5, 6, 21].
– Plain Abdominal Radiographs: Supine abdominal X‐rays may show dilated bowel, but may not detect air‐fluid levels, a sign of obstruction. Two‐view (supine + upright or supine + lateral decubitis) X‐rays are needed to document air‐fluid levels and are recommended when there is concern for mechanical obstruction or PIPO [5]. However, dilated bowel with air‐fluid levels is not a universal feature of mPIPO, particularly in infants under two months [3, 5].
– Contrast GI Series (upper GI with small bowel follow through and contrast enema): Useful to identify anatomical abnormalities such as malrotation and microcolon, assess bowel caliber, and exclude mechanical obstruction. On a practical note, water‐soluble contrast should be used instead of barium to prevent inspissation of the contrast material.
– Small intestinal transit: Radiopaque markers do not reliably delineate segmental small bowel transit [22, 23]. Nuclear medicine studies represent more reliable approaches for assessing gastric emptying, often delayed in PIPO [24], and can provide insight to guide preferential feeding methods (jejunal feedings may be needed; in the presence of delayed gastric emptying) [25]. By tracking the movement of radiolabeled test meals through the GI tract, small bowel scintigraphy (SBS) provides a physiologic and noninvasive measure of small intestinal transit. Using scintigraphy, one study showed transit was significantly slower in people with VSCM than in people with neuropathic forms of CIPO (nCIPO), in terms of median percentage of colonic filling at 6 h, both for liquid (48% for myopathic cases vs. 83% for neuropathic cases) and solid (5% myopathic subjects vs. 65% neuropathic subjects) meals [26]. Small intestinal transit performed with a solid test meal in patients with myopathic forms of PIPO (defined by antroduodenal manometry, ADM) was slower than in those with neuropathic ADM, with a colonic filling of 1.50% (IQR 1–2) at 6 h, as compared to 8% (IQR 5–26) in neuropathic PIPO (*p* = 0.006) [26], while considering that normal colon transit time typically takes around 15–20 h in healthy children [27, 28, 29]. Hence, although pediatric data are limited, SBS shows promising utility in diagnosing and characterizing PIPO, particularly in patients with myopathic involvement [26] for whom gut invasive procedures may be discouraged.
– Novel imaging modalities using cross‐sectional imaging such as multidetector row helical computed tomography (MDCT) and cine‐magnetic resonance imaging (cine‐MRI) have been recently performed with promising results in adult series, allowing the identification of intra‐ and extra‐luminal causes of mechanical occlusion (e,g, adhesions), but there is currently limited data regarding their applicability and usefulness in pediatrics [30, 31, 32, 33]. In particular, cine‐MRI is an emerging, radiation‐free technique that enables direct and dynamic assessment of small bowel motility. Advanced software applied to cine‐MRI sequences allows quantitative evaluation of global and segmental motility patterns. Although evidence remains limited, cine‐MRI appears capable of detecting impaired small bowel contractility patterns even when conventional imaging appears normal, highlighting the potential value of cine‐MRI noninvasive radiation‐free tool for evaluating people with CIPO [34, 35, 36].
– Abdominal ultrasound: This test can show bladder volume and evaluate kidney and urinary tract problems, like hydronephrosis or megacystis. Ultrasound can also be used to monitor bladder emptying to decide on the timing of bladder catheterization [37].
– Urodynamic studies: These tests should be considered in all individuals with VSCM given that involvement of the urinary tract is common [7].
– Breath tests (H_2_ or ^13^C): These depend on bacterial metabolism of ingested sugars and are unreliable as a measure of small bowel transit in CIPO, due to the almost invariable presence of bacterial overgrowth in the small bowel [15].
– Wireless capsules may assess gastric emptying and motility, but are not yet validated as a test for people with CIPO [38].

#### 2.2.2 Histopathological Evaluation in VSCM

Most people with VSCM undergo rectal biopsies to exclude Hirschsprung disease, but these biopsies rarely lead to a histopathological diagnosis of CIPO. Full‐thickness biopsies may aid mCIPO diagnosis, but the impact on patient management is modest since findings are often non‐diagnostic [39]. In unclear CIPO cases, full‐thickness sampling is suggested only if surgery is planned for another reason (e.g., ostomy) [40]. In some forms of CIPO, light microscopy, immunohistochemistry, or ultrastructure may offer clues about disease mechanisms:

– In most people with CIPO, even with known pathogenic genetic variants (e.g., *ACTG2* variants), pathologic changes are absent or non‐specific [41, 42].
– *FLNA*‐variant X‐linked CIPO shows foci of abnormally layered small intestinal muscularis propria and lacks FILAMIN A staining [43, 44].
– Mitochondrial myopathies (e.g., mitochondrial neurogastrointestinal encephalopathy (MNGIE), Alpers syndrome (*POLG1* causative variants)) often cause multifocal atrophy of the muscularis externa and megamitochondria in enteric ganglion cells ± smooth muscle [45, 46, 47]. In one study, quantitative analysis of enteric neurons of both myenteric and submucosal ganglia demonstrated a reduction in patients with MNGIE compared to jejunal tissue from non‐MNGIE patients [48].
– The combination of myocyte degeneration and interstitial fibrosis in one or both layers of the muscularis propria, especially if diffuse, supports VSCM diagnosis [48, 49].
– Hyalinized eosinophilic inclusions in the cytoplasm of smooth muscle cells, correlating with dense filamentous aggregates, is another useful diagnostic finding. These inclusions have only been described in myopathies associated with specific pathogenic gene variants (e.g., ACTG2, Duchenne) and may be the only histopathological finding in some patients. Although they are not evident in every patient with these disorders, detection of hyalinized eosinophilic inclusions should prompt genetic testing to evaluate for VSCM gene variants [41, 50, 51].

Caveats: Histopathology of gut full‐thickness specimens from CIPO has multiple challenges. In particular, VSCM is rare, with many different etiologies. In the UK experience (Section 3.1), for example, histopathology of intestinal full thickness biopsies was available for 10 VSCM‐affected patients, but only 2 showed changes suggesting muscle involvement (e.g., vacuolation in muscularis propria in ileum and colon, intracellular α‐smooth muscle actin‐positive inclusions bodies in colon, extra muscle layer on the inner aspect of the circular muscle). Moreover, few pathologists encounter enough cases to gain expertise. Numerous non‐specific changes (e.g., secondary to chronic distension, previous surgery, improper storage, delayed or inadequate fixation) affect the microscopic anatomy of the intestinal smooth muscle and may be easily misinterpreted as pathogenic [41]. For example, the observation of no smooth muscle actin expression in the inner circular layer as correlated with visceral myopathy is controversial [52]. Despite published efforts, existing pathological classification schemes are rarely used and do not reflect recent genetic insights [49, 53, 54]. Progress is likely to require multi‐center collaboration and registries linking pathology with clinical and genetic data.

#### 2.2.3 Manometry

Manometry is a functional diagnostic test that evaluates the integrity of motor patterns across different segments of the gastrointestinal tract (esophagus, stomach, proximal small intestine, colon, and anorectum) providing essential insight into the neuromuscular mechanisms underlying gastrointestinal dysmotility. Among these, ADM is currently advocated as the most discriminating investigation for supporting the diagnosis of CIPO as well as suggesting pathophysiology and possible management options [15, 55]. ADM allows both qualitative and quantitative assessment of foregut motor function by measuring intraluminal pressure in the gastric antrum and proximal small intestine. By assessing the coordination, propagation, and strength of intestinal contractions, ADM is able to distinguish different subtypes of PIPO, namely myopathic and neuropathic forms. Unlike neuropathic forms—where gut contractions show normal amplitude but are disorganized and uncoordinated—myopathic PIPO/CIPO generally shows normal phase organization of the migrating motor complexes (MMCs) and postprandial responses, but with markedly reduced contraction amplitudes (< 20 mmHg). These low‐amplitude contractions reflect impaired smooth muscle contractility [56, 57]. The clinical implications of identifying a myopathic manometric pattern are significant. Myopathic PIPO is often associated with a poorer prognosis, higher dependence on parenteral nutrition (PN), and limited response to prokinetic therapy [6, 58, 59]. Unlike nPIPO, where disorganized activity may still respond to agents that enhance neural transmission or coordination, the mechanical failure of the intestinal wall in myopathy limits therapeutic options. Children with myopathic patterns are less likely to tolerate enteral feeds. Thus, ADM findings can guide decisions regarding the need for gastrostomy, jejunostomy, or parenteral support [60, 61].

We note, however, that in the UK cohort, 5 of the 9 subjects whose genetic testing showed variants that cause VSCM had ADM patterns suggesting mixed neuropathic‐myopathic bowel dysfunction, while 2 had neuropathic changes and 2 had purely myopathic changes. This demonstrates the complexity of defining disease mechanisms in people with CIPO.

One problem is that ADM is a technically demanding test requiring specialized equipment and expertise in interpretation. A recent study highlighted that significant variability exists in ADM protocols among pediatric centers [62] suggesting that universal standardized guidelines are needed to ensure uniformity in the performance and interpretation of these studies, to enhance interobserver agreement, and allow multicenter comparisons and research. Recent efforts have introduced scoring systems and enhanced protocols to improve diagnostic accuracy and allow better discrimination between myopathic and neuropathic patterns. These tools are being refined to correlate manometric findings with histopathological diagnoses, though more data from larger populations are needed for validation [63]. ADM findings can be further complemented by colonic and esophageal manometry, especially if symptoms extend beyond the small bowel. In fact, colonic motor abnormalities—including absent high‐amplitude propagating contractions (HAPCs), impaired gastrocolonic reflexes, and a complete lack of colonic contractility—are common in children with PIPO [64]. Similarly, esophageal manometry may reveal motor abnormalities, indicating diffuse foregut involvement.

## 3 Monogenic Forms and Genetic Diagnosis of VSCM

Historically, VSCM was characterized based on manometric profiles and histopathology in the context of suggestive imaging and clinical findings. However, genetic testing, such as whole exome or whole genome sequencing, is now a primary diagnostic tool. Most cases of mPIPO are caused by pathogenic or likely pathogenic variants in genes encoding proteins involved in the smooth muscle contractile apparatus, notably *ACTG2* (gamma enteric smooth muscle actin), *ACTA2* (alpha smooth muscle actin), *MYH11* (myosin heavy chain 11), *MYLK* (myosin light chain kinase), *LMOD1* (leiomodin 1), *MYL9* (myosin light chain 9), and *FLNA* (filamin A) (for review [65, 66]). MYH11 pulls on actin filaments to generate force in smooth muscle cells; MYLK phosphorylates the MYL9 regulatory subunit of myosin to activate myosin ATPase‐induced cross‐bridge cycling to contract cells. LMOD1 nucleates actin filament formation, and FLNA is an actin filament crosslinking protein. Among these genes, *ACTG2* causative variants account for nearly 50% of genetically confirmed cases of VSCM, which are often *de novo*. Familial forms of VSCM show mostly autosomal dominant inheritance and high penetrance.

In the French pediatric cohort (2016–2024), 20 different *ACTG2* pathogenic or likely pathogenic variants were identified in 38 patients from over 160 families tested (unpublished data). In line with literature [17, 18, 66, 67], the most frequent variants were *ACTG2* p.R257C (11 patients), p.R257H (5 patients), and p.R40C (3 patients). Nearly all variants were heterozygous. Notably, 36 of the 38 variants were either *de novo* or dominantly inherited, while only two exhibited recessive transmission. Seven *ACTG2* variants remained of uncertain significance. Recurrent *ACTG2* variants that occur at arginine residues R40, R178, and R257 account for nearly 50% of the molecularly diagnosed individuals. These arginine missense changes occur at CpG dinucleotides that are hotspots for C to T transitions. Their pathogenic molecular mechanisms have been explored in biochemical studies (see Section 5.2.5 below).

Together, these genetic insights refine the classification and pathogenesis of VSCM and support the development of novel diagnostic strategies and prognostic tools. However, many patients with clinically suspected VSCM still lack a molecular diagnosis, underscoring the need for continued gene discovery and functional validation.

### 3.1 Focus on the UK Cohort

Recent data from the UK cohort, collected at the National Specialized PIPO Diagnostic Service at Great Ormond Street Hospital (GOSH) between 2012 and 2023, have provided complementary insights into the genetic landscape of VSCM. Out of 21 VSCM patients, 4 were affected with clinically diagnosed (not assessed via genetic test) MMIHS since birth, 3 with clinically diagnosed VSCM, and 14 had confirmed genetic variants. Among these, 13 patients carried *ACTG2* variants (Table 1), while 1 patient was found to harbor a compound heterozygosity for two variants in the MYH11 gene, a whole gene deletion and a missense variant (c.2366G>A‐p.R789G), both inherited by healthy parents. A similar condition [68] was reported to cause MMIHS, which also reflects the phenotype of the child, characterized by antenatally diagnosed polyhydramnios and bilateral pelvi‐calyceal dilatation, distended abdomen from birth, megaureter and megacystis requiring intermittent catheterization, global developmental delay, and malrotation.

Of the 13 *ACTG2‐*variant patients, 9 were females and presented a median age of symptom onset of 1 month. Nine distinct *ACTG2* variants were identified: c.769C>T (p.R257C); c.770G>A (p.R257H); c.533G>T (p.R178L); c.532C>T (p.R178C); c.533G>A (p.R178H); c.632G>A (p.R211G); c.119G>A (p.R40H); c.593G>T (p.G198V); c.338C>G (p.P113R). Most common symptoms at presentation of *ACTG2*‐variant patients were vomiting (13; 100%), abdominal distension (11; 85%), and constipation (7; 54%). The majority of these subjects (10/13; 77%) presented with urinary tract involvement (megacystis or bladder dysfunction). Ten out of 13 patients had extensive gastrointestinal motility testing (one patient had gastric and small bowel assessment only, due to colectomy), which showed pan‐gastrointestinal involvement in 6 patients (variants p.R257C, p.R257H, p.R211G, p.R40H, p.P113R), and dysmotility isolated to stomach and small bowel in 4 patients (variants p.R257C, p.R257H, p.R178H). All patients underwent ileostomy and the majority received venting gastrostomies. Specific variants in *ACTG2* (p.R257 and p.P113R) were associated with improved nutritional outcomes (oral/enteral feeding tolerance). Two patients bearing the p.R178C and p.R178L pathogenic variants died from liver failure while awaiting multivisceral transplant.

These findings corroborate the predominance of *ACTG2* variants in pediatric VSCM. In this small cohort, variable phenotypic expression was identified. Notably, certain *ACTG2* genotypes (e.g., p.R257 and p.P113R) may be associated with a better prognosis regarding feeding tolerance (majority of patients with either of two variants in the UK cohort were on enteral and/or oral feeding), suggesting a potential role for genetic variants in therapeutic stratification. In line with previously published data, poor outcome and severe presentation in the form of MMIHS was associated with variants at codon R178 [17]. Some other variants, such as p.R40H and p.G198V, also have unfavorable presentations (PN‐dependency and urinary tract involvement). Despite advancements in genetic diagnostics, prognostic markers and long‐term outcomes remain poorly defined, emphasizing the pressing need for better phenotypic characterization and collaborative international registries.

## 4 Up‐To‐Date VSCM Management

Acute treatment of severe VSCM symptoms focuses on bowel decompression, fluid/electrolyte replacement and optimization of nutrition. These goals often require invasive procedures, such as creation of an ileostomy and implementation of parenteral nutrition (PN). Once the patient is stable, chronic care shifts toward improving quality of life: relieving symptoms, promoting growth and GI motility, avoiding unnecessary surgery, and managing complications. A venting gastrostomy may be needed to reduce vomiting and facilitate oral feeding. Gastric or gastrojejunal tubes may also facilitate enteral (e.g., gastric or jejunal) feeding. Pharmacological treatments for VSCM are limited [65]. Antibiotics are used intermittently to treat small intestinal bacterial overgrowth (SIBO) including rifaximin, neomycin, metronidazole, ciprofloxacin and amoxicillin‐clavulanate. Prokinetics that promote gastrointestinal contractility and accelerate transit [69] include serotonergic agents such as cisapride (combined 5‐HT4 agonist and 5‐HT2B antagonist) [70], tegaserod (5‐HT4 agonist) [71] and prucalopride (selective 5‐HT4 agonist) [54, 72], reported in small case series to be helpful. These 5‐HT4 agonists work presynaptically to increase acetylcholine release from cholinergic enteric neurons. Acetylcholine is the primary pro‐contractile neurotransmitter in the bowel that induces smooth muscle contraction. Because acetylcholine is rapidly degraded after release by acetylcholinesterase, acetylcholinesterase inhibitors also increase acetylcholine throughout the body, which in the bowel and bladder increases muscle contractions to promote motility. Acetylcholinesterase inhibitors include oral pyridostigmine, which is reported to reduce symptoms and hospital stays. In acute exacerbations, neostigmine can be administered by intravenous infusion. After 10 days of full dosage, therapy can be changed to oral pyridostigmine without significant side effects. Erythromycin and amoxicillin‐clavulanic acid also have uncertain efficacy, although they appear to stimulate gastrointestinal motor activity but efficacy in VSCM is less well documented. Mechanism‐specific treatments (e.g., cholestyramine) may help with complications like biliary gastritis [73]. Avoiding anti‐cholinergic medicines is also recommended since this class of medicines prevent acetylcholine‐induced smooth muscle contraction.

Surgery may be helpful for diagnosis (e.g., biopsies) and decompression (e.g., gastrostomy, jejunostomy, ileo/colostomy) [74], although surgery should be used very sparingly, trying to avoid useless (and potentially harmful) resections often leading to the development of adhesions. Given the inverse relationship between bowel manipulation and postoperative motility recovery in VSCM‐affected patients, minimally invasive techniques are preferred to reduce bowel trauma.

Nutritional support is critical. Oral or enteral nutrition (EN) is recommended, when possible, but severe bowel impairment often requires parenteral nutrition (PN) at least intermittently. During acute obstructive episodes, stopping enteral feeding allows the bowel to decompress, reducing the risk of mechanical obstruction and reducing force generation needed to contract the bowel since wall tension is directly proportional to radius (according to Laplace's law). During fasting, total PN is usually necessary. In Italy, 77% of patients with VSCM rely on PN, with many receiving home [75].

Improvements in PN techniques (e.g., ethanol use inside central venous catheters) have reduced complications such as sepsis. TPN‐associated liver injury is also less common due to improvements in PN management: (1) avoiding overfeeding and reducing total lipids; (2) administering PN in an intermittent/cyclical way (avoiding continuous 24/24 h infusion); (3) resuming EN as soon as possible; (4) careful selection of the lipid formulation. In particular, the preferred lipid formulation is now considered the SMOFlipid emulsion (whose acronym derives from its composition: Soybean oil, Medium chain triglycerides, Olive oil and Fish oil) [76], due to the better omega 6‐omega6/omega3 ratio [77], the low level of phytosterol, and the high level of tocopherol. This special composition leads to reduced fat oxidation, and it is less toxic for the liver.

Bolus or intermittent EN is preferred over continuous EN infusion [78], promoting motility via hormonal and microbial pathways [78]. Natural dietetic patterns [79] may stimulate taste receptors located in the GI tract and microbiota (e.g., Short Chain Fatty Acids—SCFA promote serotonin release from enterochromaffin cells), potentially ameliorating motility. Patients with motility disorders can suffer gut dysbiosis (such as reduced Verrucomicrobia and Bacteroides strains, and increased Proteobacteria strains) which is possibly responsible for alterations in the cross‐talk between gut microbiota and enterochromaffin cells (EC) [80]. In this scenario, the use of natural foods can restore eubiosis, in particular by stimulating the growth of the strains involved in the communication with EC. Fecal microbiota transplantation (FMT) has shown promise in adult CIPO cases, resulting in symptom improvement and reduction of bowel dilatation [81].

A minority of people with VSCM may achieve long‐term improvement and be weaned off PN. After 24 months of good health with complete oral nutrition, stoma closure and bowel continuity restoration may be considered, using minimally invasive surgery [15]. Stoma closure may be combined with a total colectomy (if not performed previously) and subsequent ileo‐anal or ileo‐rectal pull‐through. When constipation represents the most bothersome symptom and medical treatment is ineffective, creation of a cecostomy or an appendicostomy for administration of antegrade enemas is generally safe and often beneficial. A minimally invasive approach for cecostomy has been proven feasible and safe in most series reported so far [82]. As a last resort, intestinal transplantation may be considered for patients with intractable symptoms or PN complications. However, outcomes remain suboptimal, with ~87% graft survival at 1 year and 56% at 5 years [83].

## 5 Advances in Basic and Clinical Research

### 5.1 Contribution of Muscle and Non‐Muscle Components to Intestinal Contraction

Smooth muscle in the bowel does not work in isolation, but instead forms a complex gap junction‐connected network called the “SIP syncytium”, which is essential for physiological motility [84]. The SIP syncytium consists of three cell types: (1) Smooth Muscle Cells (SMCs)—primary contractile cells generating tone and phasic contractions [85]; (2) Interstitial Cells of Cajal (ICC)—pacemaker cells that generate electrical slow waves and coordinate contractions such as peristalsis and segmentation, especially in the small intestine where a frequency gradient ensures distal wave propagation [86, 87, 88]; (3) PDGFRα+ cells—fibroblast‐like cells electrically coupled within the syncytium that transduce inhibitory signals [89]. ICC and PDGFRα+ cells generate spontaneous Ca^2+^ transients that regulate ion channels and modulate SMC excitability. Specifically, ICC depolarize SMCs to enhance contraction, while PDGFRα+ cells hyperpolarize to decrease SMC contraction [90]. Moreover, these contractile patterns are regulated by complex signaling from the enteric and autonomic nervous system [91, 92, 93]. ICC and PDGFRα+ cells closely associate with varicose terminals of enteric motor neurons and express receptors for excitatory (e.g., acetylcholine, and neurokinins) and inhibitory neurotransmitters (e.g., nitric oxide, purines, VIP, and PACAP). PDGFRα+ cells as they express receptors for purine and peptide inhibitory neurotransmitters, also mediate extrinsic sympathetic inhibition of bowel contraction via α‐adrenergic receptors [94, 95, 96]. Stimulation of post‐ganglionic sympathetic nerves reduces ICC slow wave frequency, which can influence the direction of propagation [97, 98]. This sympathetic input slows intestinal transit, enhancing time for absorption but potentially causing ileus or constipation, if excessive [99]. Hormones (epinephrine, corticotrophin releasing hormone, thyroid hormone) also impact bowel motility. Collectively, SIP syncytium, enteric nervous system, extrinsic innervation (sympathetic, parasympathetic, and dorsal root ganglia) and enteroendocrine cells in bowel epithelium generate motor patterns in the bowel in response to local mechanical (stretching, gentle brushing of the epithelium) and chemical signals (glucose, tryptophan metabolites, pH and others) as well as systemic signals (stress response, inflammation) that impact motility. These patterns include: (i) Peristalsis, driven by ICC pacemakers, for movement of contents (transit); (ii) Segmentation, arising from interactions between multiple pacemakers, aiding mixing and absorption and resulting in a waxing and waning amplitude of the slow wave [100]. This physiology suggests that intermittent increases in the severity of pseudo‐obstructive symptoms in CIPO could occur because of changes in SMC, ICC, PDGFRα+ cells, enteric neurons, enteric glia, enteroendocrine cells, sympathetic and parasympathetic neurons, hormonal signals and in response to inflammation. Pro‐inflammatory stimuli (e.g., respiratory infections) also often increase symptom severity.

### 5.2 Insights in Molecular Mechanisms Underlying Disease Pathogenesis

**FIGURE 1:**
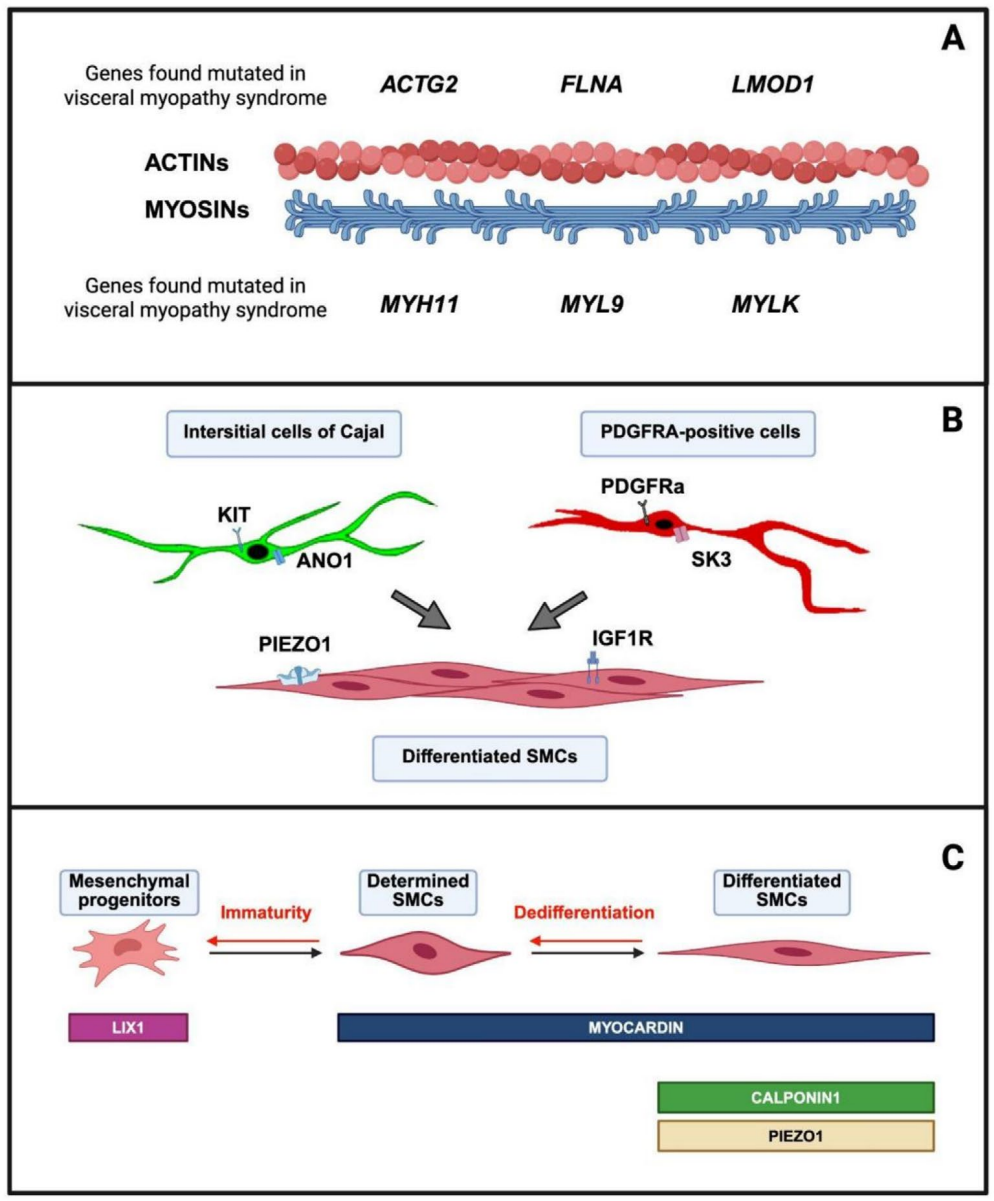
VSCM components. (A) The complex interaction of the SIP syncytium with SMCs represents the trigger of gut motility. In VSCM, the muscle component is impacted. (B) Genes of the smooth muscle contraction pathway whose variants are currently associated with VSCM onset. Most of them are tightly involved in the acto‐myosin complex. These include ACTG2, which is the most recurrent gene causative of VSCM. In the most severe forms of the disease, its variants seem to express dominant negative effects in filament polymerization. (C) SMC development and plasticity might play a role in VSCM onset, given their influence in balancing SMC contraction ability.

Disease pathogenesis for VSCM remains partially unclear. The role of the following processes, molecules, and/or mechanisms was discussed during the IFVM2024 and it is summarized in Figure 1.

#### 5.2.1 The Complex Interactions of the SIP Syncytium With SMCs

To create a resource for human SIP syncytium gene expression and identify novel genes and pathways relevant to bowel motility disorders, human single‐nucleus RNA sequencing data from 10,749 SIP syncytium nuclei (5572 SMC, 372 ICC, and 4805 PDGFRα+) from 15 individuals have been analyzed [101]. While visceral myopathy research has focused on SMCs, understanding bowel motility requires studying SMCs within the broader SIP syncytium context. This is valuable because gut dysfunction in visceral myopathies likely arises from disrupted interactions among various cell types as noted above.

Three hypotheses may explain SIP syncytium involvement in visceral myopathies: (1) SIP syncytium cells share common precursors, at least in mouse models [102, 103, 104], so variants in SMC precursor genes may also impair ICC and PDGFRα+ development. (2) Mature SIP syncytium cells express some of the same genes. For example, several proteins whose variants cause VSCM (MYH11, ACTA2, ACTG2) are expressed not only in SMCs but also in subsets of ICC and PDGFRα+ cells; (3) SMC dysfunction may lead to secondary functional changes in ICCs and PDGFRα+function over time. Additional findings include differential expression of mechanosensitive ion channels (e.g., PIEZO2, TMEM150C) in ICCs and PDGFRα+, indicating potential ENS‐independent responses to stretch.

PDGFRα+ cells also express higher levels of structural ECM (collagens and versican), nonstructural ECM (such as fibronectin, laminin, proteoglycans), and ECM remodelers (such as matrix metalloproteinases) compared to SMCs and ICCs, suggesting a role in tissue architecture. We know that bowel wall fibrosis occurs in response to inflammation and obstruction. An intriguing hypothesis to explain persistent diminished bowel motility in visceral myopathies would be a shift in PDGFRα+ cells toward a synthetic (ECM‐producing) state. This increased ECM gene expression after pathologic events would cause fibrosis, increasing stiffness of the bowel wall, fundamentally altering bowel motility.

#### 5.2.2 Mechanisms Controlling SMC Development and Differentiation

During development, the gastrointestinal tract forms from a primary tube of mesoderm and endoderm. The mesoderm develops into digestive mesenchyme, which differentiates into tissues like the submucosa and musculature, including SMCs, PDGFRα+ cells, and ICC. SMC differentiation occurs in two stages. First, mesenchymal progenitor cells express isoforms of smooth muscle actins (αSMA; γSMA; SMC determination). Later, SMCs specialize by producing contractile proteins like CALPONIN (SMC differentiation). Unlike many fully differentiated cell types in the adult body, such as skeletal muscle cells, SMCs are not terminally differentiated. Instead, they possess a remarkable ability to transdifferentiate, transitioning between a differentiated, quiescent contractile state and a highly proliferative, synthetic phenotype in response to various internal or external cues. While this plasticity is essential for maintaining muscle tissue homeostasis during both perinatal and postnatal development, aberrant plasticity has been associated with digestive motor disorders, particularly CIPO [105]. This highlights the critical need for developmental studies to uncover the mechanisms regulating SMC plasticity, which could enhance our understanding of CIPO.

In this context, a screen to search for genes with high expression at the earliest stages of stomach development was conducted, leading to the identification of the Limb Expression 1 gene (*LIX1*), which is, to date, the only known marker of digestive mesenchymal progenitors [106]. Silencing LIX1 expression during GI development impeded SMC differentiation, leading to decreased cell proliferation and diminished transcript levels and activity of the Hippo transducer YAP1 [106]. LIX1, a protein of 282 amino acids, remains relatively underexplored in terms of characterization. Its arthropod counterpart Lowfat has been found to interact with the atypical cadherins Fat and Dachsous, which play pivotal roles in regulating the Hippo pathway. LIX1 is localized in the mitochondria of stomach mesenchymal progenitors. LIX1 silencing impairs the stability of OPA1 and PHB2 specifically in mitochondria, which conducts to a structural remodeling of the cristae in the mitochondria and leading to decreased mitochondrial reactive oxygen species (mtROS) production [107, 108]. These findings highlighted LIX1's role in regulating SMC plasticity during development and in controlling mitochondrial functions, including respiration and production of mtROS. More recently, it was discovered that, while *LIX1* is typically expressed only in mesenchymal progenitors in the developing stomach during fetal life, it is highly upregulated in SMCs of CIPO patients (unpublished data). Based on these findings, a potential link between mitochondrial function and the ability of SMCs to undergo phenotypic switching [105], which is consistent with the observation that CIPO has been associated with primary mitochondrial defects [10], might involve the expression and functional role of LIX1.

From a basic science perspective, it would be interesting to explore whether the enteric nervous system (ENS) develops correctly in VSCM (see Section 2.2.3). The ENS is formed by neural crest derived progenitors that migrate within the developing intestinal wall, to colonize its entire length and establish a fully functional innervation [109, 110, 111, 112]. Myenteric plexus neurons located between circular and longitudinal muscle layers primarily regulate bowel motility, while submucosal plexus neurons regulate epithelium, immune cells and vascular function in response to local stimuli. At present it remains unknown whether and if so, to what extent, bowel innervation is affected because the muscle layers are abnormal.

Recent data on *FLNA* suggest isoform‐specific effects in gut development [44, 113]. Specifically, frameshift deletions affecting exon 2 disrupt the long *FLNA* isoform, predominantly expressed in intestinal smooth muscle. Functional models, including human intestinal smooth muscle cell lines bearing *FLNA*‐causative variants and a zebrafish knockout model of the long *FLNA* isoform, demonstrated impaired smooth muscle contractility, altered intestinal motility and abnormal intestinal elongation without altering enteric neuron number in 5‐day‐old zebrafish [44], implicating *FLNA* as essential for myogenic, rather than neurogenic, intestinal function. Interaction with the ENS to stimulate muscle growth or contractile strength is a possible route to explore, especially now that more information is emerging related to how food, and specific nutrients, activate the ENS circuitry. Distinct groups of nutrients (sugar, short chain fatty acids, amino acids) trigger the activation of selected groups of neurons in the mouse intestinal wall. Using Ca2+ imaging and Wnt1|GCaMP mice, in which all components of the ENS express a Ca2+ indicator, Fung et al. [114] monitored activity in selected neurons embedded in both the submucosal and myenteric nerve layers. Further exploration of circuit activation is ongoing. This information might be helpful to explore strategies, in which specific nerve activity triggered by nutrients might be manipulated to enhance muscle activity or stimulate muscle proliferation.

#### 5.2.3 Proinflammatory Effect of Mechanical Stressors

Changes in smooth muscle function might also be induced by mechanical stimuli, which are likely to be abnormal in people with pseudo‐obstruction. The hypothesis that pathologic mechanical stress can induce human intestinal SMC to change their gene expression profile and experience phenotypic class switching was tested in vitro. SMCs seeded on anisotropic electrospun nano‐fibrous scaffolds were exposed to uniaxial cyclic stretching as a mechanical stressor or left unstretched for comparison. RNA sequencing revealed that this stress altered the expression of 4537 genes and led to a shift to a more synthetic and proinflammatory phenotype. This transition was characterized by increased expression of proinflammatory cytokines and synthetic smooth muscle genes, including extracellular matrix components. Further analysis suggested that many of the differentially expressed genes encode secreted ligands that could influence not only HISMCs but also other cells in the bowel wall. These findings underscore the rapid phenotypic plasticity of HISMCs in response to mechanical stress, potentially contributing to bowel diseases such as myopathic intestinal pseudo‐obstruction, which is characterized by symptom exacerbations following distension‐inducing events [115].

#### 5.2.4 Mechanotransduction in Gut

Both development and aging lead to physical changes in many organs. For example, aging brings increasing stiffening of several tissues, which collectively contribute to functional decline in the brain, heart, and vasculature, bladder, and GI tract. Unraveling these mechanisms may provide insights into visceral myopathies, which often involve molecular elements associated with mechanotransduction. The stiffening of the gut wall with age was correlated with delays in GI transit and urinary incontinence, also demonstrating a novel function of the mechanosensitive ion channel PIEZO1 [116] in translating substrate stiffness to smooth muscle reprogramming away from contractile toward synthetic phenotype. A detailed biophysical study of primary fibroblasts obtained from a VSCM patient carrying a very rare situation of double variant in *PIEZO1* was also presented, comparing the mechanical and morphological phenotype of *ACTG2* variants with such samples. While cells bearing the *ACTG2* p.R257C causative variant exhibit reduced traction forces and increased motility [117], cells with *PIEZO1* pathogenetic variants were almost immobile with strong adhesions to the substrate (unpublished data, Vassalli).

#### 5.2.5 Inside γ‐Smooth Muscle Actin (ACTG2)

##### 5.2.5.1 Dynamic Characteristics of Pathogenic Variants of Smooth Muscle Actins In Silico

Actin, a key protein in eukaryotic cells, supports cell motility, division, shape, and muscle contraction. It exists as monomeric (G‐actin) and polymeric (F‐actin) forms, with six human isoforms, including smooth muscle‐specific γSMA and αSMA. Variants in actin genes can cause disorders like VSCM and thoracic aortic aneurysms (TAAD). Both γSMA and αSMA share structural similarities, with specific causative variants (p.R257C/p.R258C) destabilizing actin filaments, leading to disease phenotypes. Molecular dynamics simulations were employed to assess structural effects of γSMA‐p.R257C and αSMA‐p.R258C variants in both G‐actin and F‐actin, totaling 1.5 μs simulation time [118]. Despite their sequence similarity, the two isoforms showed different variation‐induced structural responses in simulations, possibly explaining distinct disease outcomes. Here's a detailed breakdown of the key findings: (a) Conformational Effects: p.R257/8C variants alter actin flexibility, stability, and dynamics, impacting polymerization and ATP hydrolysis. (b) Filament Stability: γSMA‐p.R257C causes filament bending and disrupted hydrogen bonding, especially at filament ends. αSMA‐p.R258C induces milder structural changes but leads to a more compact filament. (c) Pathology: Disrupted filament integrity and actin's ability to properly polymerize or interact with binding proteins likely underlie VSCM and TAAD mechanisms. (d) Therapeutic Potential: A druggable pocket near residues R257/8 could be targeted to modulate actin dynamics. Overall, γSMA‐p.R257C was predicted *in silico* to mainly cause filament fragmentation, while αSMA‐p.R258C was predicted to promote actin filament depolymerization—both impacting smooth muscle function differently and offering distinct therapeutic angles.

##### 5.2.5.2 Biochemical Mechanisms of Causative ACTG2 Variants

A new method enabled production of recombinant human actin with native folding and post‐translational modifications. Wild‐type γSMA and four VSCM‐associated ACTG2 variants (p.R40C, p.R148C, p.R178C, and p.R257C) were analyzed in vitro to explore how each variant disrupts actin biochemistry [119]. These studies showed that the p.R178C causative variant led to rapid degradation post‐purification suggesting instability of the protein tertiary structure. This correlates with early‐onset, severe VSCM (MMIHS) phenotypes commonly seen with this variant in vivo. The p.R148C variant, which is associated with milder later‐onset disease, disrupted ACTG2 binding to the gelsolin domain (G4‐G6). This same actin region binds other proteins, suggesting p.R148C may disrupt interactions with other actin‐binding proteins. The p.R40C causative variant failed to polymerize and interfered with WT‐actin and leiomodin‐1 interactions, showing dominant‐negative behavior. The p.R257C is the most commonly reported ACTG2 variant causing VSCM. It polymerized faster than WT but formed unstable filaments that broke quickly under the pulling force of smooth muscle myosin heads. The ACTG2 p.R257C also exhibited dominant negative effects on filament stability when incorporated into mixed filaments (50% WT/50% variant protein) in vitro.

##### 5.2.5.3 Functional Impact of *ACTG2* Variants

Functional studies further support these insights. The *ACTG2* gene codes for gamma enteric smooth muscle actin (γSMA), a tissue smooth muscle specific actin isoform [120]. In MMIHS, the most severe *ACTG2*‐related phenotype, it has been demonstrated [67] that specific γSMA variants (p.R40C, p.R63Q, p.R1478S, p.R178H, p.R178C, and p.R178L) impair actin polymerization and that the ACTG2 p.R178 variant reduces collagen gel contraction in U2OS cells. Similar contraction impairments have been found in patient‐derived fibroblasts [117]. These data support a dominant‐negative effect and suggest a block in early smooth muscle lineage commitment, associated with baseline TGF‐β activation and aberrant αSMA expression (unpublished data).

#### 5.2.6 The Long Non‐Coding RNA CARMN

Long noncoding RNAs (lncRNAs), defined as transcripts longer than 200 nucleotides with no apparent protein‐coding potential, outnumber protein‐coding genes and have been shown to play critical roles in various physiological and pathological processes [121].

Using a data mining approach to interrogate multiple independent omics datasets, a novel lncRNA, cardiac mesoderm enhancer‐associated non‐coding RNA (*CARMN*), has been identified as the most abundantly expressed lncRNA in visceral SMCs in both humans and mice. Unexpectedly, both germline and SMC‐specific inducible deletion of *Carmn* in mice, led to premature lethality due to intestinal pseudo‐obstruction, driven by downregulation of numerous smooth muscle contractile genes, including *MYLK*, a known CIPO‐associated gene [122, 123]. Mechanistically, CARMN promotes the smooth muscle contractile gene program by activating the essential transcriptional complex SRF/MYOCD through direct interaction with MYOCD [122, 124].

These findings establish CARMN as indispensable for bowel smooth muscle function, identifying the first lncRNA with a critical role in regulating GI motility. This raises the possibility that variants in *CARMN* impairing its expression or function may underlie certain cases of idiopathic CIPO. Future studies are therefore warranted to determine whether reduced *CARMN* expression or loss‐of‐function variants occur in people with VSCM.

## 6 3D Models for Studying VSCM

The creation of disease models is essential in studying ultra‐rare disorders like VSCM. At the conference, several 3D models were presented. One promising approach involves the evaluation of contractile ability of human intestinal smooth muscle ex vivo in an oxygenated organ bath. Preliminary studies show quantitative assessment of mCIPO muscle contraction correlates with loss of gut contraction in people affected with VSCM, showing a weaker contractile response (reduced force and displacement) compared to controls (unpublished data, Viti). Despite the limited sample availability due to disease rarity and surgical constraints, this functional tool may preserve disease‐specific features that cannot be fully recapitulated in 2D cellular or molecular models.

To overcome tissue scarcity, alternative 3D models are being developed by combining in vitro intestinal smooth muscle cells, ICC pacemaker cells, and enteric neurons [125]. Using a novel serum‐free Muscularis medium and a 3D electrospun scaffold, researchers achieved long‐term spontaneous contractions and three natural‐like motility patterns (“clamshell,” “oceanwave,” “jellyfish”) [37, 126]. This bioengineered muscle demonstrated the ability to mix fluids and physically break down artificial intestinal contents, marking a breakthrough in gut tissue engineering. Subsequent analyses of contractility, cellular morphology, and transcriptome profiles confirmed that both the Muscularis medium's biological components (crucial for ion‐transport functions) and the scaffold's physical cues (which regulates cell–cell communication) are vital for creating a fully functional intestinal muscle patch.

Induced pluripotent stem cell‐derived human intestinal organoids (HIOs) offer another in vitro strategy for modeling development, microbiota interaction, and drug testing. Although promising, HIOs still lack mature visceral smooth muscle structure (both the alignment of cells in the smooth muscle and the muscle bilayer) [127, 128, 129] although this problem can be overcome by growing organoids in a confined culture system followed by transplantation into immunocompromised rats [130]. A material‐based model using a gut phantom was also presented. This strategy aims to restore motility and addresses the loss of intestinal wall tone via mucoadhesive magnetic nanoparticles encapsulated in pH‐responsive alginate hydrogel and functionalized with chitosan. These encapsulated nanoparticles were shown to be safe and effective in a 3D cell model, improving motility without crossing the intestinal barrier or compromising nutrient absorption [131]. To support testing, a duodenum‐mimicking phantom with accurate mechanical properties and a mucosal layer was fabricated using Polydimethylsiloxane (PDMS) and surface‐functionalized with 3% v/v aminopropyltriethoxysilane (APTES) for mucin binding [132].

## 7 Family Experience

The term Patient Advocacy Organizations or Public Aid Organizations (PAO) is used to represent third sector voluntary agencies or charitable bodies and, in general, patient‐focused entities that provide support to patients living with one or more chronic health conditions, and their caregivers [133]. PAOs roles and activities range from advocating for patient needs, to creating connections with health care institutions with specific interests in their diseases. With this purpose, they facilitate connections among affected individuals, promote awareness and understanding of the disease, raise financial support for research, and lobby at the local and central Government level. The role of PAOs is particularly important when their activities are focused on patients with rare diseases, such as CIPO. These conditions present significant challenges for both patients and healthcare providers, as lack of knowledge even among specialists [8] and lack of interest from profit‐oriented research institutions, make them particularly difficult to diagnose, manage, and treat. Patients with CIPO face serious health and social problems on a daily basis: (1) occlusive episodes expose them to the risk of repeated, often unnecessary, and potentially life‐threatening surgical procedures even after firm diagnosis is established [9]; (2) since doctors and surgeons are at times not even aware of the existence of this disease [8], it may take years before a definitive diagnosis is established [9, 15]; (3) oral nutrition can be often challenging in adults and children and the condition may be confused with psychiatric problems like anorexia nervosa; (4) home parenteral nutrition is necessary in many patients to grant sufficient nutritional support and this requires special training and skills of caregivers to prevent serious complications [15, 134]; (5) symptoms are disabling during disease exacerbations and can be severe even between episodes, disrupting family and social relationships; (6) CIPO has a huge economic burden for families of affected individuals regardless of the age of disease onset, since patients often cannot work and extended hospital stays make work difficult for caregivers. PAOs play a crucial role in addressing these challenges by providing a platform for these patients, their families and their needs. PAOs are very active in Europe [135] and those focusing on CIPO are no exception. *Uniti per la PIPO* (www.unitiperlapipo.it), *POIC e dintorni* (https://poic‐e‐dintorni.org/) the *Voluntary Organization of the Italian Group for Intestinal Pseudo‐obstruction* (GIPsI OdV; www.gipsi‐cipo.it), and the French‐speaking *Association des POIC* (https://association‐poic.fr/) are European nonprofit organizations specifically active in this area. The *International Foundation for Gastrointestinal Disorders* (IFFGD iffgd.org/gi‐disorders), the *MMIHS Foundation* (https://www.mmihs.org/) and the *World Visceral Myopathy Foundation* (https://wvmf.org/) in the United States provides support and information for people affected by diverse GI disorders and is particularly active in lobbying and raising funds for research in chronic digestive disorders.

Collaboration among nonprofit organizations might significantly enhance patient advocacy and foster a stronger, unified voice for research and policy change, ultimately benefiting both the social well‐being and healthcare outcomes of individuals affected by this rare condition. During the conference, PAOs emphasized the urgency felt by families living with CIPO. Here are some of the most impactful sentences: “Your time is not our time”, “You take off the CIPO coat when you leave your laboratories and your hospitals. Our children wear it every hour, every day”. This call to action highlighted the urgent need to accelerate and deepen research and clinical care, driven not only by scientific curiosity, but also by the experiences and hopes of families waiting for breakthroughs. When asked about their priorities regarding main research goals, the PAOs listed, in addition to the obvious goal of curing the disease, identifying drugs that promote motility, the desire for something to relieve symptoms, and a non‐invasive way to monitor health and functioning of the gut.

In conclusion, PAOs are essential for individuals with rare diseases such as CIPO. Through support, education, awareness promotion, research funding, and policy advocacy, these organizations help to create a more informed, supportive, and effective healthcare environment for patients. Even more importantly, they provide hope for a future in which better treatments and, ultimately, cures are within reach. As rare disease advocacy continues to grow, the power of these organizations will remain a driving force in improving the lives of patients and their families.

## 8 Conclusions

This manuscript highlights the clinical and molecular complexity of VSCM as a rare but severe disease. Currently, genetic diagnoses can be made in about half of all individuals with VSCM and provide the most definitive method to define disease etiology. Genetic diagnoses also facilitate prognosis and individualized treatment planning, although phenotypic variability is wide, even among patients harboring identical pathogenetic variants. While imaging can exclude mechanical causes of bowel obstruction and may show dilated bowel typical of pseudo‐obstruction, these tests do not define disease mechanisms. Manometry may distinguish mCIPO (defined by weak muscles) from nCIPO (defined by normal strength of muscle contraction but abnormal motility patterns). However, some cases of genetically defined mCIPO are not recognized by manometry. Histopathology also often shows nonspecific changes, even in genetically confirmed cases. The management of severe VSCM involves bowel decompression via ileostomy and gastrostomy, nutritional support including PN, and symptom control, with limited pharmacologic options. Minimizing surgery is important due to its negative impact on motility. Some patients recover enough to discontinue parenteral nutrition and undergo reconstructive surgery; intestinal transplant remains a last‐resort option. The role of the autonomic nervous system warrants further exploration. Understanding the role of gut microbiota changes and possible restoration of eubiosis represents another future area of research.

Because VSCM is rare, establishing a wide, international, collaborative scientific community of clinicians and researchers cooperating with PAOs and families is essential to advance knowledge of different forms of the disease. The establishment of an international registry is crucial to enhance understanding, enable genotype–phenotype correlations, and guide future therapeutic research. Collection and sharing of images from histopathology and functional assessment can foster the development of stronger and standardized diagnostic guidelines specifically devoted to VSCM.

The development of disease models, including ex vivo tissues, 2D in vitro cellular models, 3D bioengineered muscle and organoids, as well as animal models, is crucial for allowing studies to trigger a better understanding and treatment of VSCM. In this perspective, the development of cell and tissue biobanks coupled to a disease registry can support novel basic‐research studies.

PAOs and families play a vital role in supporting affected individuals, funding research, and raising awareness, offering hope for improved treatments and eventual cures. Advocating policy changes is difficult for such ultra‐rare diseases, although policymakers' involvement is crucial to produce relevant changes at a national health system level. PAO influence is essential for enhancing patient care and outcomes, and it serves as a positive trigger to speed up research about this very real, genetically heterogeneous group of diseases known as VSCM.

## Author Contributions

All authors provided substantial contributions to the conception/design of the work, drafted the work/revised it critically for important intellectual content, and finally approved the version to be published.

## Funding

The IFVM204 conference (https://ifvm2024.ge.ibf.cnr.it/) was economically supported by: ‘Gipsi Odv’, ‘Poic e dintorni’, ‘Association de POIC’, ‘Uniti per la PIPO’ patients’ associations. Fimatho. Association Française contre les Myopathies (AFM‐Telethon N°28,600) granted to P.d.S.B. Italian Research Ministry (PRIN 2022 ref. 2022JKEBB8) granted to F.V. IRCCS Giannina Gaslini (Ricerca Corrente) granted to I.C. Moreover, Aurogene srl, Chiron Biotech, Clinisciences, Baxter companies sponsored the event.

## Conflicts of Interest

The authors declare no conflicts of interest.

## Data Availability

The authors have nothing to report.
